# Transcriptomic Analysis of *Aedes aegypti* in Response to Mosquitocidal *Bacillus thuringiensis* LLP29 Toxin

**DOI:** 10.1038/s41598-018-30741-x

**Published:** 2018-08-23

**Authors:** Khadija Batool, Intikhab Alam, Songqing Wu, Wencheng Liu, Guohui Zhao, Mingfeng Chen, Junxiang Wang, Jin Xu, Tianpei Huang, Xiaohong Pan, Xiaoqiang Yu, Xiong Guan, Lei Xu, Lingling Zhang

**Affiliations:** 1 State Key Laboratory of Ecological Pest Control for Fujian and Taiwan Crops, College of Life Sciences, Key Lab of Biopesticides and Chemical Biology, MOE, Fujian Agriculture and Forestry University, 350002 Fuzhou, Fujian PR China; 2 Key Laboratory of Genetics, Breeding and Comprehensive Utilization of Crops, Ministry of Education, College of Crop Science, Fujian Agriculture and Forestry University, 350002 Fuzhou, Fujian People’s Republic of China; 3 Division of Cell Biology and Biophysics, University of Missouri – Kansas City, Kansas City, MO 64110 USA

## Abstract

Globally, *Aedes aegypti* is one of the most dangerous mosquitoes that plays a crucial role as a vector for human diseases, such as yellow fever, dengue, and chikungunya. To identify (1) transcriptomic basis of midgut (2) key genes that are involved in the toxicity process by a comparative transcriptomic analysis between the control and *Bacillus thuringiensis* (Bt) toxin (LLP29 proteins)-treated groups. Next-generation sequencing technology was used to sequence the midgut transcriptome of *A. aegypti*. A total of 17130 unigenes, including 574 new unigenes, were identified containing 16358 (95.49%) unigenes that were functionally annotated. According to differentially expressed gene (DEG) analysis, 557 DEGs were annotated, including 226 upregulated and 231 downregulated unigenes in the Bt toxin-treated group. A total of 442 DEGs were functionally annotated; among these, 33 were specific to multidrug resistance, 6 were immune-system-related (Lectin, Defensin, Lysozyme), 28 were related to putative proteases, 7 were lipase-related, 8 were related to phosphatases, and 30 were related to other transporters. In addition, the relative expression of 28 DEGs was further confirmed through quantitative real time polymerase chain reaction. The results provide a transcriptomic basis for the identification and functional authentication of DEGs in *A. aegypti*.

## Introduction

The mosquito *Aedes aegypti* is one of the most dangerous diseases-causing vector and is found in most tropical areas worldwide. However, Africa is considered the ancestral place of this species^1^. *A. aegypti* plays a crucial role as a vector for human diseases, such as yellow fever, dengue^2^, chikungunya, and Zika fever^3–6^. The dengue virus (DENV) infects about 100 million patients every year due to complexity and existence of different serotypes (DENV1, DENV2, DENV3, and DENV4)^7,8^. The chikungunya virus (CHIKV) causes chikungunya fever with severe pain in the joints of infected patients for many years^9^. A previous study demonstrated that CHIKV continues to exist as a co-infection with dengue^10^. The Zika virus (ZIKV) also spreads through the blood feeding of *A. aegypti* and causes serious health issues globally^6^ and is becoming extremely predominant in Brazil resulting in microcephaly among newborns. The virus affects the growth of brain and forms cranial calcifications^11^. Recently, an outbreak of Zika virus occurred around Central America, South America, and the Caribbean, and is associated with prenatal brain malfunction^12^. Currently, due to the lack of antiviral drugs and vaccines against the arbovirus, the main strategy to control mosquito-borne diseases is through vector control^13,14^.

*Bacillus thuringiensis* (Bt) plays an important role in pest control management and in public health and is widely used^15–17^. *B. thuringiensis* subsp. *israelensis* (Bti) produces Cry toxins that have been used for mosquito control. These toxins are highly toxic to mosquitoes, such as *Aedes*, *Anopheles*, *Culex, Mansonia*, and *Simulium* larvae^18^. A new subspecies of Bt, LLP29, was reported for the first time from the leaves of *Magnolia denudata*, and has been found to be highly toxic to mosquitoes. It is a promising Bt strain to control mosquitoes because of its high toxicity and short life cycle^19,20^. The Cry toxins are mainly involved in (1) protoxin solubilization, (2) protoxin proteolytic activation by specific proteases, (3) interaction between putative receptors and active toxins, (4) oligomerization of toxin, and (5) insertion to epithelial cells. Thus, forming pores in the plasma membrane of midgut cell, and eventually leading to cell death^16,17^. The use of Cry toxins against insect pests depends on the interaction between Cry toxins and other receptors, such as alkaline phosphatase (ALP), aminopeptidase-N (APN), and ATP-binding cassette (ABC) transporters^16,21–24^. Therefore, it is important to understand the interaction of Cry toxins with other midgut proteins.

As toxin binding is partially dependent on receptor glycosylation^25^, proteins that interact with carbohydrate-recognition domains (CRD) might affect toxicity. Furthermore, the transcriptomic sequencing is a useful method to determine the composition and function of DEG genes of many complex insects, including *Drosophila melanogaster*^26^, *A. aegypti* and *Anopheles gambiae*^27^, *Bombyx mori*^28^, and *Maruca vitrata*^29^. In the present study, next-generation sequencing technology was used to sequence the transcriptome of *A. aegypti* exposed to LLP29 protein, and successfully built a transcript database; In addition, putative insecticide resistance transcript, immune-system related transcript, stressed-related transcript, detoxification-related molecules, and selected DEGs were confirmed through quantitative real time polymerase chain reaction (qPCR). Furthermore, the present study provides valuable information that can be used to develop new genetics-based strategies and novel molecular tools to control *A. aegypti*.

## Results and Discussion

### Sequence analysis and functional annotation

LLP29 is highly toxic to many mosquitoes, such as *A. aegypti*, *A. albopictus* and *Culex quinquefasciatus*^19,20^. Meanwhile, because of fewer plasmids and short life cycle, it is good for reducing the cost of Bt application and has potential as a good biocontrol agent in mosquitoes^20^. In order to further understand the transcriptomic basis of midgut and its main functional genes after Bti LLP29 infection, transcriptomic analysis of *A. aegypti* in response to LLP29 was carried out in the present research, other than only single Cry toxin, such as Cry11Aa^30^. After complete sequencing and quality check, a total of 9.32 Gb of clean data were obtained. The Q30 base percentage of each sample was above 85.77%, which showed reliable base identification and limited base error detection. We generated 40,678,664 and 33,418,822 raw reads from the control and *Bacillus thuringiensis* (Bt) toxin (LLP29 proteins)-treated groups and 29,434,409, 24,268,858 reads were mapped to reference genome of *A. aegypti*. A total of 17130 unigene sequences were identified in the midgut of *A. aegypti* (Table S1). These unigenes were compared by BLAST with those in Swiss-Prot^31^, GO^32^, NR^33^, COG^34^, and KEGG databases^35^. A total of 16358 (95.49%) unigenes were functionally annotated by BLAST searches against all databases (Table 1). Furthermore, with the development of sequencing technique, 574 new unigenes that not present in any published annotation were identified in the transcriptome of *A. aegypti*, including 391 unigenes that were functionally annotated through different databases (Table 2). They will contribute further to the transcriptome of *A. aegypti* and bioinformatic approaches, and greatly benefit the knowledge of Bt mechanism on immune response^36^.

**Table 1:** Functional annotation of all assembled unigenes.

Annotated_Database	Annotated_Number	length < 300	300 < = length < 1000	length > = 1000
COG_Annotation	5010	53	1515	3442
GO_Annotation	11360	317	4236	6807
KEGG_Annotation	6811	237	2346	4228
Swissprot_Annotation	9762	246	3512	6004
nr_Annotation	16357	1027	6548	8782
All_Annotated	16358	1027	6549	8782

**Table 2:** Functional annotation of assembled new unigenes.

Annotated_Database	Annotated_Number	length < 300	300 < = length < 1000	length > = 1000
COG_Annotation	51	0	12	39
GO_Annotation	195	2	79	114
KEGG_Annotation	151	3	60	88
Swissprot_Annotation	126	2	45	79
nr_Annotation	390	7	166	217
All_Annotated	391	7	167	217

**Figure 1:**
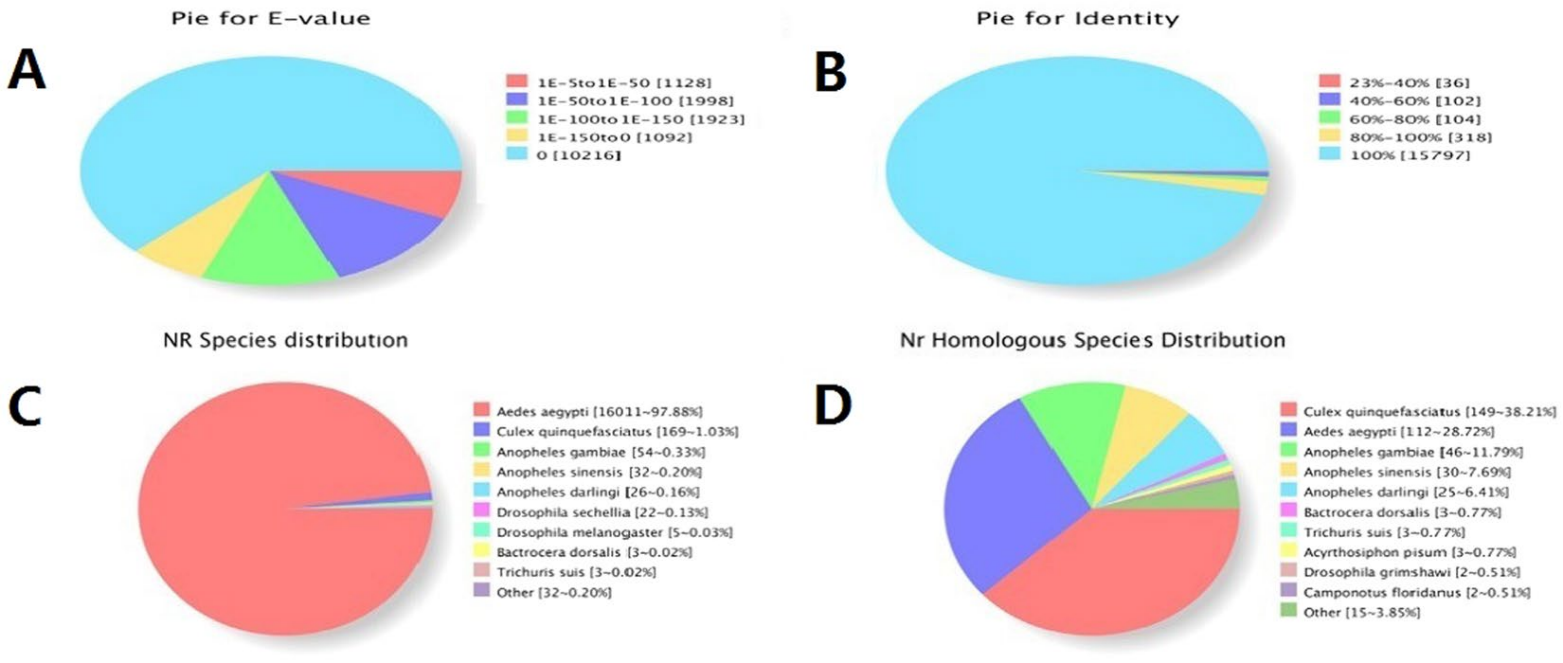
The Nr annotation of all unigenes and new unigenes. (**A**) Shows the E-value distribution; (**B**) is the similarity distribution; (**C**) shows the top species distribution of all unigenes; (**D**) shows the top species distribution of all new unigenes.

As shown in Fig. 1, among all the annotated unigenes, 10216 (62.4%) unigenes showed a high homology (E-value 0), which specifically matched the NR database (Fig. 1A). The identity comparison showed that 16115 (98.5%) unigenes have more than 80% identity with insects (Fig. 1B). Further, 16325 unigenes were distributed into 9 species (Fig. 1C). Approximately, 16011 unigenes (97.8%) were annotated to the top-hit species *A. aegypti* and other top species including, *Culex quinquefasciatus* (169, 1.03%), *Anopheles gambiae* (54, 0.33%), *A. sinensis* (32, 0.20%), *A. darlingi* (26, 0.16%), *Drosophila sechellia* (22, 0.13%), *D. melanogaster* (5, 0.03%), *Bactrocera dorsalis* (3, 0.02%), and *Trichurissuis* (3, 0.02%); while 32 unigenes were distributed into other species (Fig. 1C). The homologous species distribution of 390 newly identified unigenes is shown in Fig. 1D. The top-hit species were *C. quinquefasciatus* (149, 38.21%), *A. aegypti* (112, 28.72%), *A. gambiae* (46, 11.79%), *A. sinensis* (30, 70.69%), *A. darlingi* (25, 6.4%), *B. dorsalis* (3, 0.77%)*, T. suis* (3, 0.77%)*, Acyrthosiphon pisum* (3, 0.77%)*, D. grimshawi* (2, 0.51%), *Camponotus floridanus* (2, 0.51%), while 15 other unigenes were distributed into other insect species (Fig. 1D).

**Figure 2:**
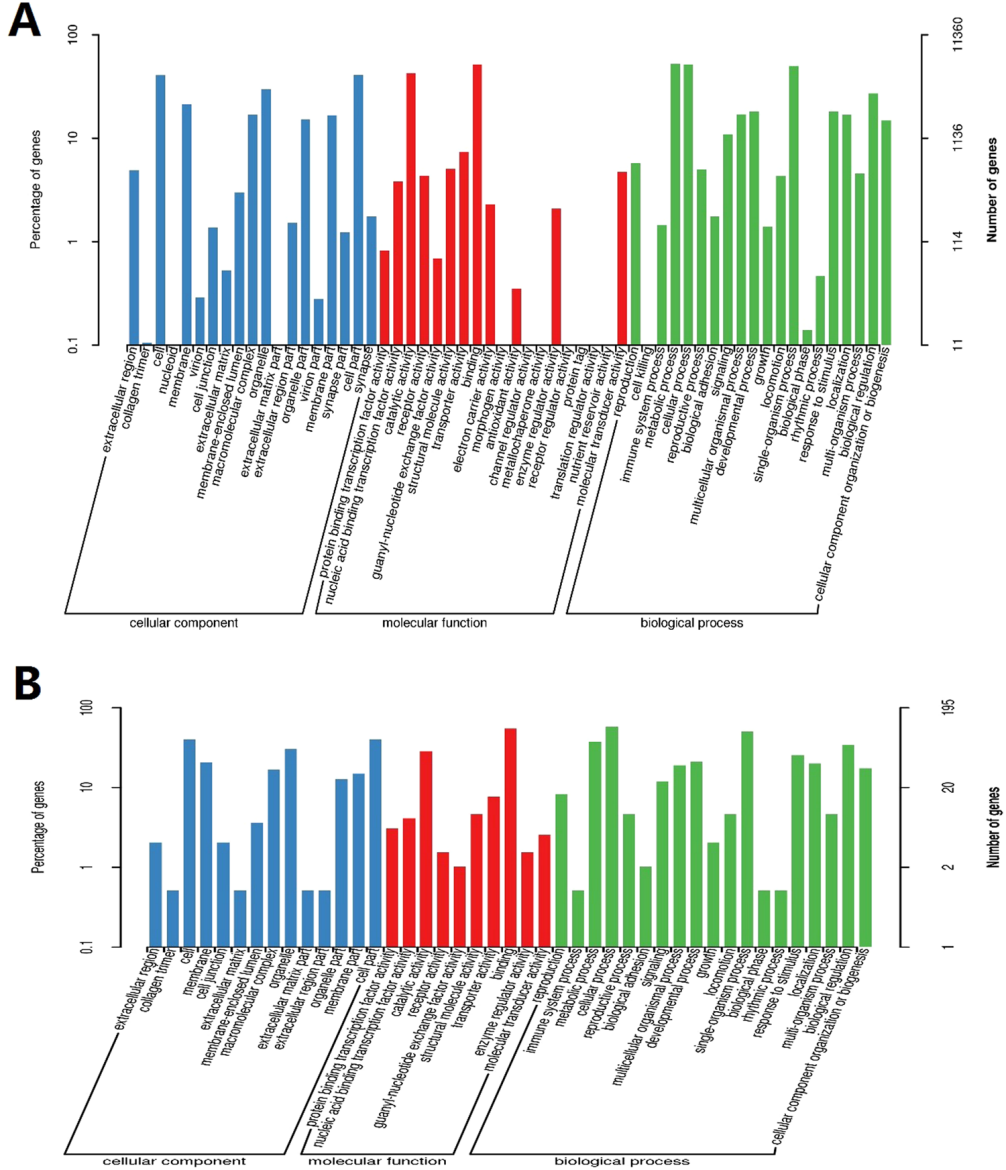
The gene ontology (GO) annotation of consensus sequences. (**A**) shows all unigenes; (**B**) shows newly identified unigenes from mid gut of *Aedes aegypti*.

**Figure 3:**
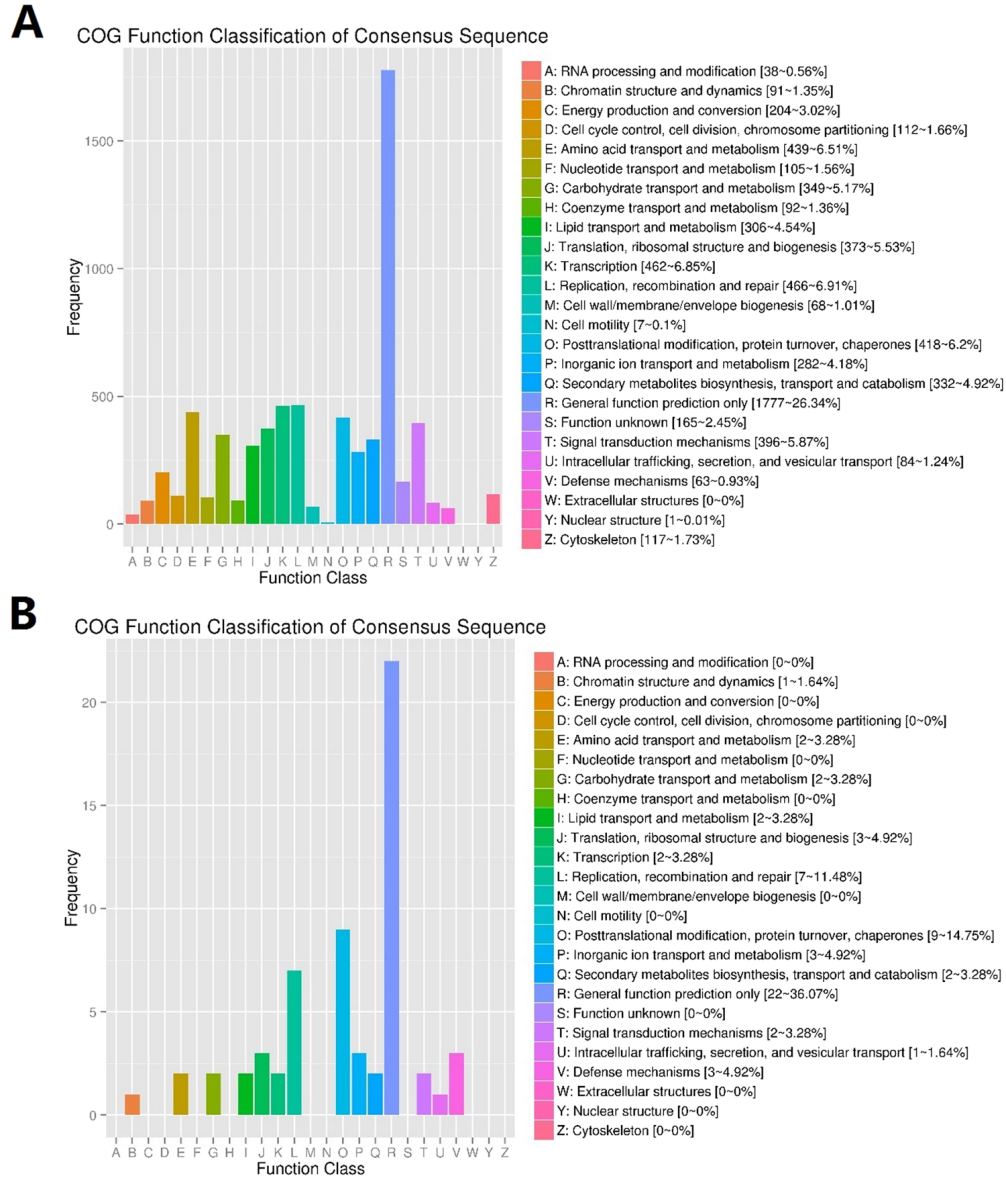
The Cluster of Orthologous Groups (COG) classification. (**A**) Shows the all unigenes; (**B**) shows the newly identified unigenes from mid gut of *Aedes aegypti*.

To understand the predicted function of all unigenes, GO analysis was also performed. The genes were classified into three precise GO categories, including biological process, cellular component, and molecular function. A total of 11360 unigenes, including 195 new unigenes were annotated from the GO database and classified into biological process (8556, 75.31%), cellular component (6311, 55.5%), and molecular function (9779, 86.08%) unigenes (Fig. 2A,B; Table S2). They were further subdivided into different subcategories including 20 subcategories under biological process, and 19 subcategories under cellular component and molecular function. In the biological process, the most abundant were metabolic (5950, 69.5%), cellular (5874, 68.6%), and single organism processes (5692, 66.5%) (Fig. 2; Table S2). Cellular component included cell part (73.58%), cell (4634, 73.42%), and organelle (3385, 53.63%); and molecular function included binding (5910, 60.43%) and catalytic activities (4866, 49.75%) (Fig. 2; Table S2). The top two subcategories in all three levels were similar to those reported in other species^37,38^. For further functional classification of all the unigenes, the COG database was used. About 5010 unigenes, including 51 new unigenes, were classified into 24 different COG categories (Fig. 3A,B; Table S3). Among the COG categories, the major class was the ‘general function prediction only’ (1777, 35.4%), followed by ‘replication, recombination and repair’ (466, 9.30%) and ‘transcription’ (462, 9.22%) (Fig. 3, Table S3).

Furthermore, all unigenes were functionally classified based on the KEGG pathway analysis. A total of 6811 unigenes were annotated from the KEGG pathway database, and were distributed into 258 KEGG pathways (Table S4). The highly abundant KEGG pathways were RNA transport (ko03013) with 168 unigenes, ribosome (ko03010) with 162 unigenes, purine metabolism (ko00230) with 158 unigenes, protein processing in the endoplasmic reticulum (ko04141) with 151 unigenes, spliceosome (ko03040) with 136 unigenes. Ninety nine unigenes were related to three different xenobiotic-metabolism pathways related to cytochrome p450 (ko00980, ko00982, and ko00983) and other 28 unigenes were related to the innate immune system, such as toll-like receptor (TLR) pathways (ko04620), and reactive to Gram-positive bacterial and fungal infections^39^. The other 32 putative unigenes were related to the TNF pathway (ko04668), while the IMD signaling pathway was considered a homolog of TNF pathway based on the records of the KEGG map and responsive on Gram-negative bacteria^39,40^. Further, the JAK/STAT pathway was found to be related to 26 unigenes, and the JAK/STAT pathway (ko04630) is involved in multiple developmental processes and immune responses^40^, such as inhibition of microbial infection and repair of septic damage^40,41^.

### Differential gene expression

**Figure 4:**
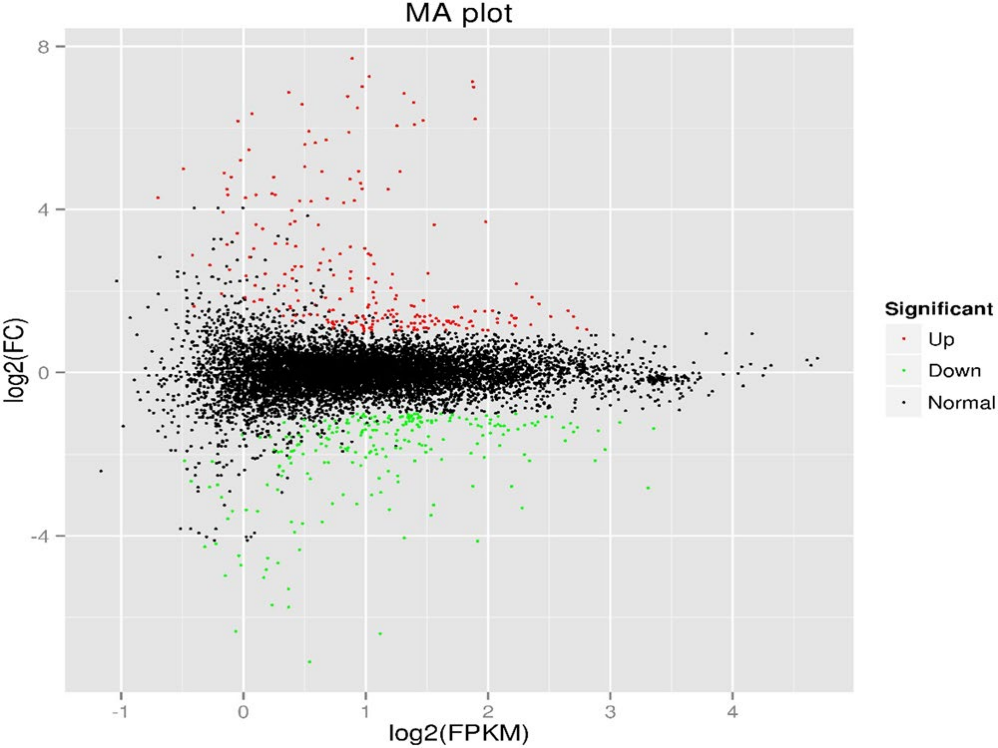
MA plot of differentially expressed genes (DEGs). The log2 fold-change indicates the mean expression level for each gene. Each dot represents one gene, and black dots represent no significant differences between control and LLP29 protein treated groups. Red and green dots represent significantly up- and down-regulated genes, respectively.

To identify specific target genes that affect different biological processes, the gene expression profiles were compared to examine changes in gene activity among the control and Bti LLP29 protein-treated groups. To identify significant differences in gene expression, FPKM with fold change >2 and FDR <0.01 were used as the thresholds. Based on this parameter, 457 unigenes were found to be differentially expressed in the midgut of *A. aegypti* (Table S5). Among the differentially expressed genes, 226 were upregulated and 231 were downregulated in response to Bti toxin. The number of upregulated unigenes was nearly similar to the number of downregulated unigenes (Fig. 4; Table S5). In this study, several homologous genes potentially implicated in the immune response to LLP29 toxin were found to be highly differentially expressed and similar results were also obtained in other previous studies in response to insecticides or single toxin, such as Cry11Aa^30,42–44^.

**Figure 5:**
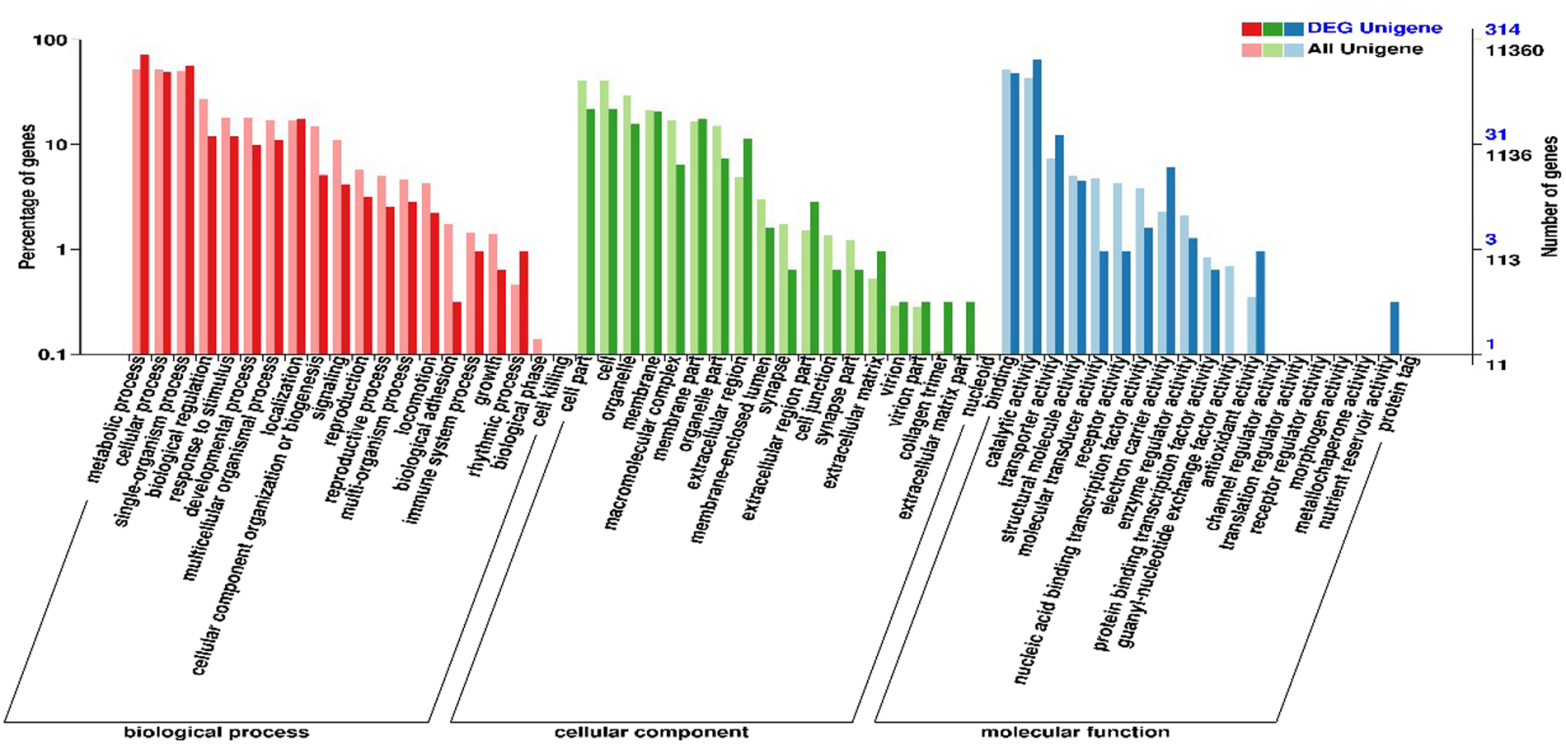
The gene ontology (GO) annotation of differentially expressed unigenes between control and LLP29 protein treated groups.

**Figure 6:**
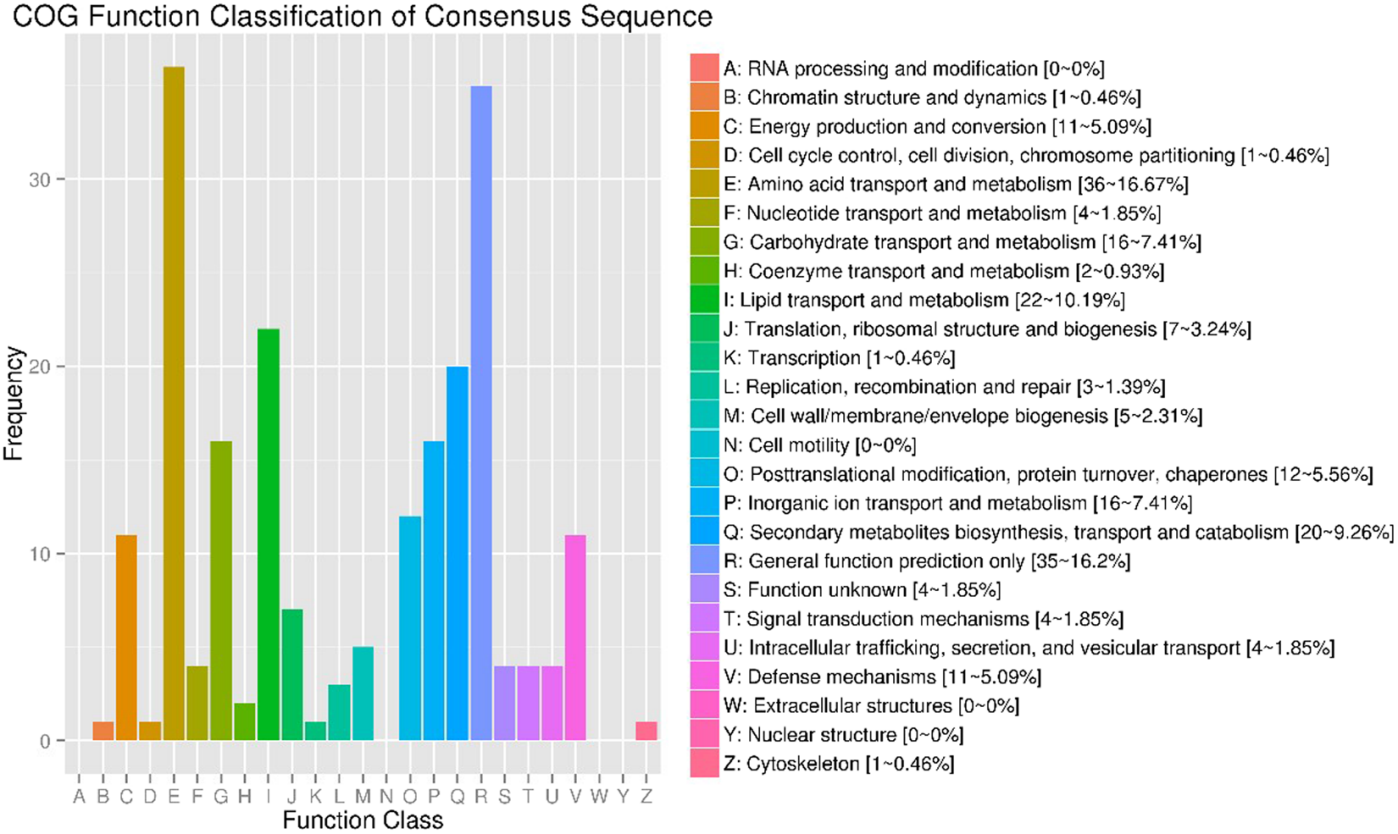
The Cluster of Orthologous Groups (COG) classification of differentially expressed unigenes.

**Figure 7:**
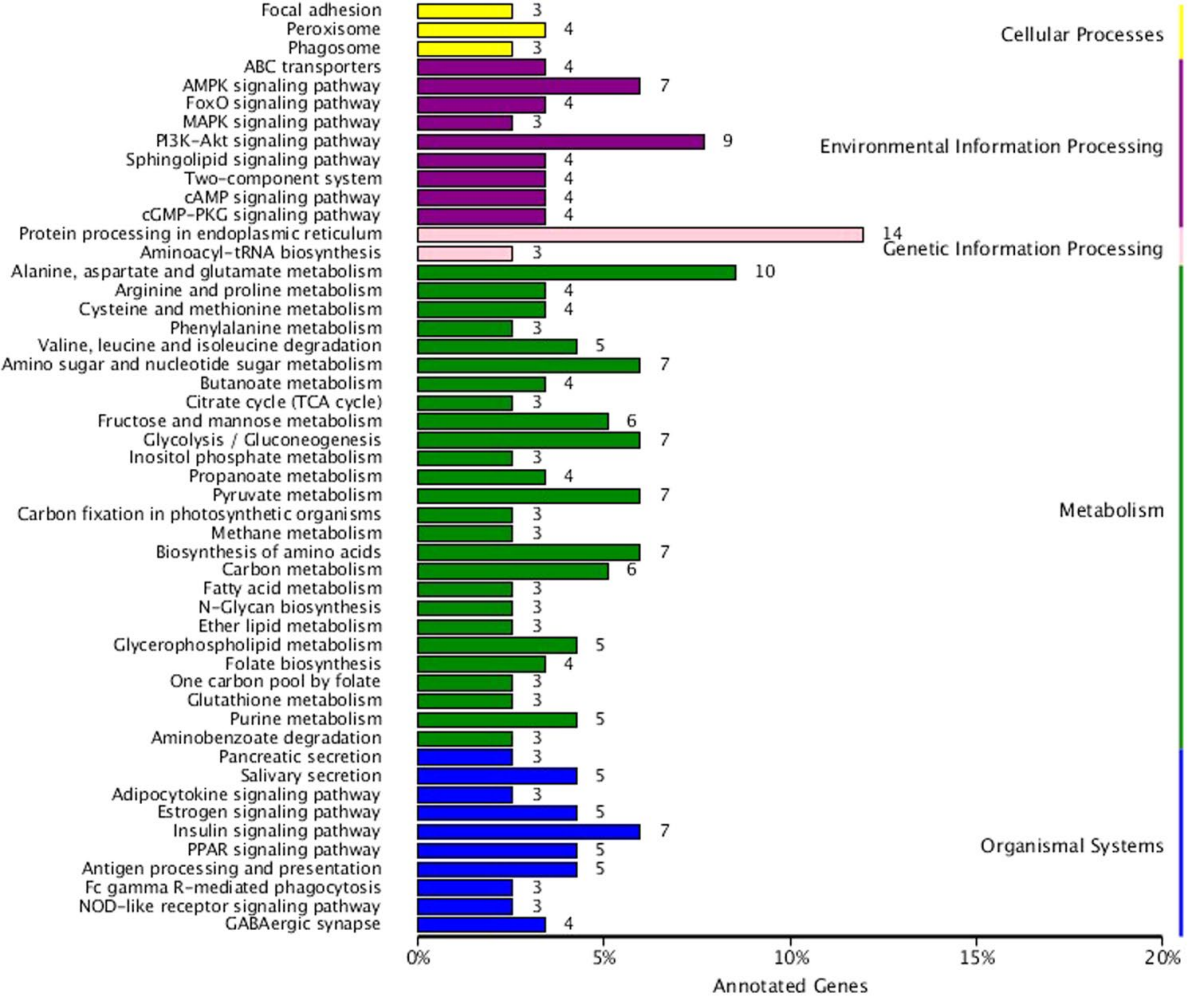
The top Kyoto Encyclopedia of Genes and Genome (KEGG) pathways for differentially expressed unigenes.

A total of 442 DEGs were successfully annotated by BLAST searches with at least one database (Table S6). Based on the GO analysis (314, 71.04%), the DEGs were annotated and classified into 3 categories—(1) biological processes (270, 61.08), including metabolic (227, 84.07%), cellular (155, 57.4%), and single organism processes (178, 65.9%), (2) cellular component (50, 47%), including cell part (68, 45.33%) and cell (68, 45.33%); and (3) molecular function (293, 66.28%), including binding (151, 51.53%) and catalytic activities (201, 68.6%). Most of these categories were enriched (Fig. 5; Table S7). To identify the COG function, 165 DEGs were identified, which were further classified into 21 main categories basing on the COG function classification. The amino acid transport and metabolism group (16.69%) is the highest class, followed by the general function prediction (16.2%) (Fig. 6). According to the KEGG pathway analysis, 172 differential genes were identified that are involved in various biological pathways of *A. aegypti* (Fig. 7; Table S8). Further, these 172 DEGs were annotated from the KEGG pathway database, and the most enriched unigenes were mapped into 50 different KEGG pathways. They were further categorized into 5 classes: metabolism was the most represented class with 61 DEGs, including 26 different metabolism pathways which might play a role in LLP29 toxin resistance. Among them Alanine, aspartate and glutamate metabolism (ko00400), followed by Amino sugar and nucleotide sugar metabolism (ko00520), Glycolysis/Gluconeogenesis (ko00010), Pyruvate metabolism (ko00620), Biosynthesis of amino acids (ko01230), and Carbon metabolism (ko01200), a highly enriched pathways. This was followed by the organismal systems class with 27 DEGs, including 10 different system pathways, the most enriched was the Insulin signaling pathway (ko04911), followed by PPAR signaling pathway (ko03320), Estrogen signaling pathway (ko04915), Antigen processing and presentation (ko04612), Salivary secretion (ko04970) and GABAergic synapse pathway (ko04727). In environmental information processing class with 25 DEGs, including 9 different signal transduction pathways, the most enriched pathway was the PI3K-Akt signaling pathway (ko04151), followed by AMPK signaling pathway (ko04152) and MAPK signaling pathway (ko04010). However, signal transduction pathways also been reported to have an important role in response to Cry11Aa toxin^45,46^. Within genetic information processing class with 10 DEGs which included two different pathways, the protein processing in endoplasmic reticulum (ko04141) was the most highly enriched pathway and the other pathway was aminoacyl-tRNA biosynthesis (ko00970). The cellular processing class had 10 DEGs, including three pathways, focal adhesion (ko04510), peroxisome (ko04146), and phagosome (ko04145) (Fig. 7).

### Specific protein types related to multidrug resistance and immune system

Based on the BLASTx results and searches for target genes related to protein families with specific multidrug resistance/defense, 68 multidrug resistance protein genes were identified in the midgut transcriptome of *A. aegypti*. The specific domain related to multidrug resistance was confirmed using Pfam and Interpro databases^47,48^. Among these unigenes, 11 ATP-binding cassettes (ABC) transporter unigenes were differentially expressed, including 5 unigenes that were upregulated and 6 unigenes that were downregulated. However, most of the (ABC) transporter unigenes were reported downregulated in the intoxication with Cry11Aa toxin to *A. aegypti*^30,44^. Furthermore, the highly expressed genes were also searched, and the results revealed 4 ABC transporter unigenes whose FPKM values were >100 (Table S9). Except for Cry11Aa, there are more mosquitocdial toxins harbored in LLP29, such as Cry4, Cry10, Cyt1 and Cyt 2, etc. With different toxins infection, the complex immune response of ABC transporter unigenes needs further study.

The cytochrome P450 is another multidrug resistance protein involved in many physiological functions, such as resistance to insecticides and hormone metabolism^49,50^. The association of cytochrome P450 with propoxur, dichlorvos, and pyrethroid resulted in insecticide resistance in *C. pipiens* mosquito^51^. A total of 175 cytochrome P450 unigenes were identified in the midgut of *A. aegypti*. Among these unigenes, 11 cytochrome P450 unigenes were differentially expressed. Interestingly, all these unigenes were upregulated, while they were downregulated in other reported studies of *A. aegypti*, such as in Bti tolerant *A. aegypti* strains^42^, and Cry11Aa toxin resistant *A. aegypti*^43^, chemical and biological insecticide strains of *A. aegypti*^44^,and intoxication of Cry11Aa toxin to *A. aegypti* larvaes^30^, and these monooxygenases play an important role in the degradation of insecticides^52^. Furthermore, they also respond to Cry1Ab protoxin in Lepidopterans, *C. fumiferana* and *M. sexta*^53^. Eighteen highly expressed unigenes of cytochrome P450 with FPKM value >100 were identified in the present study (Table S9).

The cytosolic matrix glutathione S-transferases (GSTs) have been reported as important detoxification-related protein in the metabolism of insecticides^54^. In insects, the cytosolic matrix GSTs are classified into six groups (delta, epsilon, omega, sigma, theta, and zeta) according to the sequence similarity, immunoreactivity, specificity of substrate, and sensitivity to inhibitors^55,56^. Meanwhile, delta and epsilon classes are very unique in insects^57^. In the present study, 26 GST unigenes were identified. Among them, two GST unigenes were differentially expressed, including one upregulated and one downregulated while the same gene was found up regulated in response of Cry11Aa toxin to *A. aegypti* larvaes^30^. However, it is important to notice that we found similar results as coded by other previous studies^42,44^, while six unigenes were highly expressed with a FPKM value > 100 (Table S9). The identification GST unigenes in the midgut transcriptome of *A. aegypti* is very important. Glutathione S-transferase plays a major role in the detoxification of secondary metabolites and insecticides^58,59^, thus providing high resistance to insecticides^60^.

Studies have reported that insect carboxylesterases are involved in xenobiotic metabolism, and have been classified into 13 clades^61^. In the present study, 46 putative carboxylesterases/ juvenile hormone esterases were also identified. Among these, 9 unigenes were differentially expressed, including 4 upregulated and 5 downregulated DEGs while 5 unigenes were highly expressed (FPKM value > 100) (Table S9). However most of the unigenes related to carboxylesterases were downregulated at the exposure of LLP29 toxin at 14 hours of treatment. It is notable that most of the carboxylesterases related unigenes were also down regulated in response to insecticides or toxins in other reported studies^30,43,44^.

**Figure 8:**
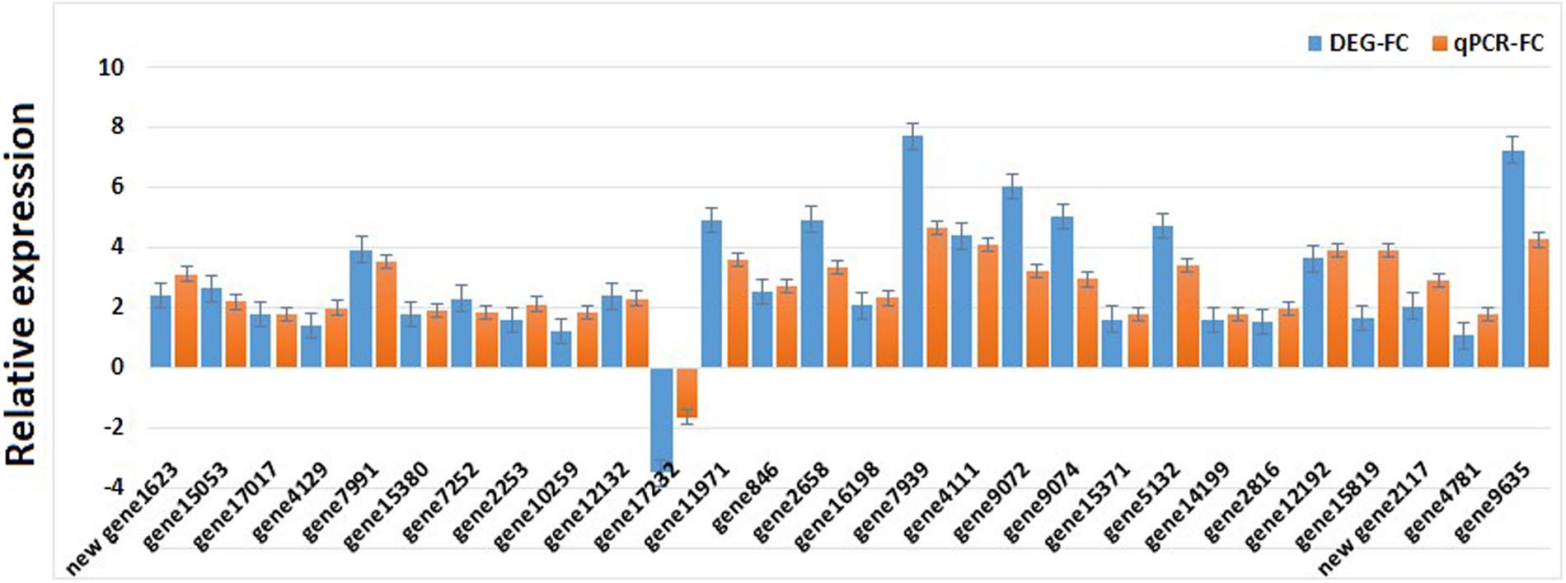
qRT-PCR analysis of DEGs and comparison to transcriptomic data. The unigenes represent by x-axis and the scale of relative expression showed by y-axis.

Lectins are a class of carbohydrate binding proteins ubiquitously expressed in plants, animals, bacteria, and viruses^62^. The lectin is a major constituent of humoral immune system of insects, and is involved in the process of self/non-self-recognition. Interestingly, lectin proteins can recognize specific carbohydrate structures, and can bind to the cell surface sugars to agglutinate the cells^63^. Some insect lectins recognize polysaccharide chains on the surface of pathogens, and probably are involved in self-defense. In the present study, 47 lectin genes were identified in the midgut of *A. aegypti*, including G-specific type and C-type lectin genes. Only one differentially expressed lectin unigene, which was upregulated against the Bti LLP29 protein, was identified. Five highly expressed lectin unigenes with FPKM value > 100 (Table S9) were also identified. Further, defensin proteins have been reported to exhibit a strong innate immune response to a wide range of pathogenic bacteria^64^. In the present study, four defensin-related unigenes were identified; however, only one defensin-related unigene was differentially expressed and was downregulated in response to the Bti LLP29 protein (Table S9). Lysozyme is another antimicrobial enzyme that takes part in innate immunity. It catalyzes the hydrolysis of specific 1,4-beta linkages between N-acetylmuramic acid and N-acetyl-D glucosamine residues in peptidoglycan, thus lysing the bacterial cell^65^. In the present study, 9 unigenes related to lysozyme, including one upregulated and two downregulated DEGs (Table S9). These putative immune response unigenes were further validated by RT-qPCR (Fig. 8).

### Transcript-related digestion, lipid/fatty acid, phosphatases, and other transporter proteins

In the present study, the digestion-related genes, including various protease groups, were examined. The results revealed that 28 differentially expressed unigenes were putative proteases, including serine protease, trypsin, chymotrypsin, venom serine protease, carboxypeptidase, and other metalloproteinases. The serine proteases are involved in Cry protoxin activation via proteolytic elimination of peptide fragments^66^. In addition, 45 highly expressed unigenes related to a protease with FPKM value of >100 were identified (Table S10). Earlier studies have reported that chitinases catalyze the hydrolysis of chitin. As chitin is present in the cell wall of many green algae and fungi, and in the exoskeleton of numerous crustacean and insects^67^, chitinases can be used in the biocontrol of plant pathogenic fungi and insects^68^. In the present study, 4 DEGs related to chitinases, including 3 upregulated unigenes, were identified in the midgut of Bti toxin-treated *A. aegypti*. Five chitinase-related unigenes highly expressed with FPKM value > 100 were identified (Table S11). The chitinases related genes were also reported in previous studies in response to insecticides or toxins^42–44^. However, no chitinase related gene was reported in the single Cry11Aa toxin to *A. aegypti* larvaes^30^.

An alpha amylase-related unigene has been identified as a receptor for Cry4B and Cry11A in *A. albimanus*^69^. However, in the present study, 2 upregulated alpha amylase-related unigenes were identified that might play an important role in Bti resistance while 7 alpha amylase-related unigenes were highly expressed (Table S11). Furthermore, 5 unigenes related to AMP dependent ligases, including 4 upregulated and one downregulated DEGs and two highly expressed unigenes with FPKM value > 100 were identified (Table S11). Further, 7 differentially expressed and 4 highly expressed unigenes (FPKM value > 100) related to putative lipases/phospholipases were identified (Table S11). Furthermore, 2 fatty acid-related unigenes were differentially expressed, while 6 fatty acid-related unigenes were highly expressed (Table S11). Three hundred and five (305) putative unigenes related to phosphatases were identified. Among these, 8 upregulated unigenes were differentially expressed, including 3 alkaline phosphatases (ALPs), 2 inositol polyphosphates, and 2 protein phosphatases (Table S11). Six (6) phosphatase-related unigenes highly expressed with FPKM value > 100 were identified (Table S11). Alkaline phosphatases have been recognized as receptors for putative Cry1Ac in *Heliothis virescens* and the lepidopteran *Manduca sexta*. Furthermore, alkaline phosphatase has been reported as a receptor of proteins Cry4B and Cry11A in *A. aegypti*^22,70–72^.

In the present study, 308 transporter protein-related unigenes were identified in the midgut of *A. aegypti* treated with Bti LLP29 protein. These included (1) 30 differentially expressed unigenes—5 sugar transporters, 7 sodium-dependent nutrient transporters, 3 amino acid transporters, 3 zinc transporters, 3 synaptic vesicle proteins, 2 mitochondrial carriers, and 7 different transporter-like genes; and (2) 13 highly expressed unigenes with FPKM value > 100 (Table S11). The genes related to lipid and fatty acid metabolism, binding, and transport have been reported to play a key role in the movement and homeostasis of lipid-related substances in the midgut^53,73^. However, based on BLASTx and Pfam search, no lipophorins were identified in the midgut by some studies^74,75^. In the present study, one triacylglycerol lipase^76^ was identified. The hemolymph of some insects contains lipoproteins that bind to xenobiotics during microbial infection^75^. Therefore, the expression of lipid-related genes might be a defense mechanism in response to Bt LLP29 toxin. The combination of lipid-related and highly expressed transporter-related unigenes suggests the role of midgut in the transportation of lipid-bound proteins and peptides.

### qPCR analysis of selected different types of related genes

To verify the differential expression values of the transcriptomic data, qPCR was used in this study. A total of 28 genes were randomly selected from different families, including multidrug resistant genes: ABC transporter (3); cytochrome P450 (3); carboxylesterase (1); juvenile hormone (1); GST (1); lectin (1); defensin (1); lysozyme (1); proteases including serine proteinase (1), trypsin (1), carboxypeptidase (1), metalloproteinase (1), and venom serine protease; lipase (2); chitinase (1); alkaline phosphatase (1); inositol polyphosphate (1); sodium transporter (2); sugar transporter (2); zinc transporter protein (1); and ammonium transporter (1). Among these, majority of the genes presented relatively similar transcriptomic and qPCR data in terms of expression (Fig. 8). While the expression of some genes, such as gene7939, gene9072, gene9074, and gene9635, was 2–3 fold higher in the transcriptomic data when compared with that in the qPCR data (Fig. 8).

## Materials and Methods

### Sample preparation

*A. aegypti* were reared in an environmentally controlled room at 28 °C and 85% relative humidity (RH) with a photoperiod of 14:10 h light/dark. The Bt strain LLP29 was isolated from leaves of Mognolia denudate Desr. (Magnoliales: Magnoliaceae)^19,20^ and were grown in nutrient broth sporulation medium containing erythromycin with final concentration of 50 ug/ml at 30 ^o^C temperature for three days, following cell autolysis, spores and inclusion protein was harvested and washed three times with distilled water and 1 M NaCl. After resuspending the inclusions were purified according to the previous report^77^. The total protein (Bti toxin LLP29 protein) of concentration 0.255 μg/mL was fed to fourth instar *A. aegypti* larvae (100 larvae/cup) in 50 ml of dechlorinated water for 14 hours. For negative control, the larvae were not treated. After exposure the severely affected and dead larvae were discarded, and the survived larvae were used for RNA extraction. Both samples were used in triplicates. Midguts were dissected from both samples carefully under stereomicroscope (VHX-5000, KEYENCE), and placed in RNA-hold (Transgen biotech.) and stored at −80 degrees until further use.

### Illumina sequencing and cDNA library

Total RNA was isolated from the midgut of the fourth-instar larvae using Trizol reagent (Invitrogen, USA). The isolated total RNA was precipitated with RNase free DNase I to remove genomic DNA using Amp Grade (Invitrogen, USA), following the instructions of the manufacturer. The quality of RNA was checked by agarose gel electrophoresis, and also by using the NanoDrop ND-1000 spectrophotometer (Thermo Scientific, USA). To prepare RNA samples, 1.5 μg RNA was used per sample as initial material. Sequence library was created through NEBNext1 Ultra™ RNA Library Prep Kit for Illumina1 (NEB, USA), according to the instruction from the manufacturer. Messenger RNA was purified from the total RNA using poly-T oligo-attached magnetic beads. Fragmentation was carried out using RNA fragmentation kit, followed by the synthesis of first and second strand complementary DNAs using M-MuLV reverse transcriptase (RNase H-), DNA polymerase I, and RNaseH. The complementary DNA fragments were end-repaired and ligated to a NEB Next Adaptor after adenylation of the 3′ ends, and then purified with the AMPure XP system (Beckman Coulter, Beverly, USA) to create a cDNA library. The library quality was assessed on the Agilent Bioanalyzer 2100 system. After clustering, the products were sequenced on an Illumina Hiseq. 2500 platform to generate 100 bp paired-end reads (Biomarker Technologies Co., Ltd., Beijing, China). The Illumina GA Pipeline (version 1.3) was used to filter the raw data to eliminate the adapter sequences, low-quality reads and empty reads.

### Bioinformatic analyses

Reads were compared and mapped to reference genome of *A. aegypti* Liverpool strain (GCA_000004015.2) by using Bowtie and topHAT2 software^78^. RSEM software was used to convert the number of reads mapping to each unigenes as FPKM (Fragments Per Kilobase of transcript per Million mapped reads) and estimated the expression level of corresponding unigenes^79,80^. Furthermore, EB Seq software was used for differential expression analysis^81^. Fragments per kilobase of exon model per million mapped reads (FPKM) of fold change >2 and false discovery rate (FDR) of <0.01 were used as the thresholds to identify significant differences in gene expression. All unigenes were compared with the Swiss-Prot^31^, Gene Ontology (GO)^32^, Non Redundant (NR)^33^, Clusters of Ortholgous Groups (COG)^34^, Kyoto Encyclopedia of Genes and Genomes (KEGG)^35^, and other databases using the BLAST software^82^. The E-value of the Basic Local Alignment Search Tool (BLAST) parameter was set at 1−5. The amino acid sequence of candidate unigene-related proteins was investigated through Pfam database to gain annotated information^47^.

### Quantitative real-time PCR validation

The selected DEGs were confirmed by (q-PCR). Total RNA from each sample was extracted as described earlier. Reverse transcription was carried out using the PrimeScript RT reagent kit with gDNA Eraser (TOYOBO). Quantitative real-time PCR was conducted on the CFX96 Real-Time System (Bio-Rad, Hercules, CA, USA) by SybrGreen method with Premix Ex Taq II (Takara, Kyoto, Japan). The qPCR cycling condition: 95 °C for 30 s, followed by 40 cycles at 95 °C for 30 s, and 60 °C for 35 s was used to confirm selected genes using specific primer and 40 s rRNA as control and efficiency values were detected at the range of 94–99% (Table S12). The qPCR product specificity was analyzed using the melting curve. The relative gene expression values were calculated by the 2- ΔΔCt method^83^.

## Conclusions

LLP29 is a highly mosquitocidal Bti strain with independent intellectual property that harbors fewer plasmids and then in shorter life cycle^19,20^. In order to further know Bt mechanism and provide the theory base of mosquito biocontrol application, LLP29 was used as a novel Bti candidate, and the transcriptomic basis of midgut as well as the main functional genes of *A. aegypti* after Bt infection was searched by transcriptome Illumina resequencing in the present study. In the present study, the midgut of control and LLp29 toxin-treated *A. aegypti* mosquitoes was sequenced by Illumina sequencing. A total of 17130, including 16358 unigenes were functionally annotated by BLAST searches against various databases. Furthermore, 574 new unigenes, including 391 functionally annotated unigenes, were identified. A total of 557 DEGs, including 226 upregulated and 231 downregulated unigenes, were annotated by comparing the Bti toxin-treated and control groups. Furthermore, 442 DEGs were functionally annotated. Furthermore, specific DEGs related to multidrug resistance, immune response, stressed-related, detoxification, lipid/fatty acid, phosphatases, and other transporter proteins were identified. In addition, 28 randomly selected DEGs were further confirmed through qPCR. Majority of genes exhibited similar transcriptomic and qPCR data in terms of expression. The results of the present study are more valuable than the ones only immunity to Cry11Aa toxin in Bti and it will be useful for further research on interaction between Cry toxins and other proteins. Due to the presence of many putative proteins and these midgut proteins may compete with Cry toxin to bind with receptors and protect different receptors from toxin binding, thus altering the toxicity of Cry toxins. Further research is needed to identify the other midgut proteins that can interfere with interaction between Cry toxins and different receptors, and also to identify polysaccharides or chemicals that counterpart to neutralize the effect of these midgut proteins to increase the toxicity of Cry toxins for mosquito control.

## Electronic supplementary material

Supplementary Information

## Data Availability

The transcriptome raw data has been submitted to the National Center for Biotechnology Information (NCBI). Read data can be accessed directly in Sequence Read Archive (SRA) with accession number SRP150934.
